# Anti-psoriatic potential of nanocarrier formulated with *Deverra tortuosa* DC. and *Deverra triradiata* hochst aerial extracts: in vivo evaluation in mice

**DOI:** 10.1007/s44446-026-00086-y

**Published:** 2026-05-26

**Authors:** Reem A. Kamel, Asmaa A. Ahmed, Mohammed S. Teiama, Sabah H. Elgayed, Mohamed A. Khattab, Magda T. Ibrahim, Doaa Abouelenein, Giovanni Caprioli, Ahmed M. Mustafa, Elsayed K. El-Sayed, Fatma A. Moharram

**Affiliations:** 1 Department of Pharmacognosy, Faculty of Pharmacy, Al-Azhar University, Cairo, 11651 Egypt; 2 Department of Pharmacology and Toxicology, Faculty of Pharmacy, Helwan University, Ain Helwan, Cairo, 11795 Egypt; 3 Department of Pharmaceutics and Industrial Pharmacy, Faculty of Pharmacy, Helwan University, Ain Helwan, Cairo, 11795 Egypt; 4 Department of Pharmaceutics and Industrial Pharmacy, Faculty of Pharmacy, Galala University, New Galala City, 43713 Suez Egypt; 5 Department of Pharmacognosy, Faculty of Pharmacy, Cairo University, Cairo, 11562 Egypt; 6 Department of Pharmacognosy, Faculty of Pharmacy, 6th October University, Cairo, 11562 Egypt; 7 Department of Cytology and Histology, Faculty of Veterinary Medicine, Cairo University, Giza, 12211 Egypt; 8 Department of Pharmacognosy, Faculty of Pharmacy, Heliopolis University for Sustainable Development, Cairo, 11785 Egypt; 9 School of Pharmacy, University of Camerino, Via Sant’ Agostino 1, 62032 Camerino, Italy; 10 Department of Pharmacognosy, Faculty of Pharmacy, Zagazig University, Zagazig, 44519 Egypt; 11 Department of Pharmacognosy, Faculty of Pharmacy, Helwan University, Ain Helwan, Cairo, 11795 Egypt

**Keywords:** Psoriasis, *Deverra tortuosa*, *Deverra triradiate*, HPLC–MS/MS, Nanocream, Anti-inflammatory

## Abstract

Psoriasis is a chronic autoimmune skin disorder marked by rapid skin cell proliferation, causing red, scaly patches. Current treatments often show limited efficacy and adverse effects. The family Apiaceae is traditionally valued for its medicinal uses, particularly anti-inflammatory effects, and among its members are *Deverra tortuosa* (Desf.) DC. (*D. tortuosa*) and *Deverra triradiata* Hochst. ex Bioss. (*D. triradiata*) with recognized therapeutic value. As nanotechnology-based formulations enhance skin penetration and therapeutic efficacy, this study investigated the antipsoriatic activity of plant-based nanocreams (NCs). Phenolic content of *D. tortuosa* and *D. triradiata* aerial parts was analyzed by high-performance liquid chromatography–tandem mass spectrometry (HPLC–MS/MS). Nanoparticles were prepared with droplet sizes of 300–470 nm and a positive surface charge. Mice were divided into five groups: control (cream base), imiquimod (IMQU; 62.5 mg/day for 7 days), a commonly used inducer of psoriasis-like dermatitis, standard (tacrolimus 20 mg/kg/day, 2 h after IMQU), IMQU + *D. triradiata* NC (2% once daily, 2 h after IMQU), and IMQU + *D. tortuosa* NC (2% once daily, 2 h after IMQU). *D. tortuosa* and *D. triradiata* NCs significantly alleviated IMQU-induced systemic and skin-related psoriatic changes in mice by reducing spleen index, inflammatory cytokines, including interleukin-17 (IL-17), interleukin-6 (IL-6), and tumor necrosis factor-alpha (TNF-α), as well as nuclear factor kappa-B (NF-κB), signal transducer and activator of transcription-3 (STAT3), and Ki-67 expression. Histological analysis confirmed marked restoration of normal skin architecture, with *D. tortuosa* showing the highest therapeutic efficacy. These findings suggest that *D. tortuosa* and *D. triradiata* NCs possess promising anti-psoriatic potential, through immunomodulatory and anti-inflammatory mechanisms, with *D. tortuosa* demonstrating superior efficacy. They may serve as effective natural alternatives to conventional psoriasis treatments.

**Supplementary Information:**

The online version contains supplementary material available at 10.1007/s44446-026-00086-y.

## Introduction

Psoriasis is a chronic inflammatory condition caused by immune responses, affecting about 4.4% of the global population (Skayem et al. 2025). Although the cause is unknown, genetic and environmental factors such as trauma, infection, stress, and medications may contribute (Branisteanu et al. 2022). Clinically, it presents as well-defined, pruritic erythematous plaques with silvery-white scales on extensor surfaces and the scalp, with possible nail changes and psoriatic arthritis (Saal et al. 2025). Histological findings include epidermal hyperproliferation with keratinocyte dedifferentiation, enhanced angiogenesis, and inflammatory cell infiltration (Kamata and Tada 2023; Man et al. 2023). Stress-activated keratinocytes release tumor necrosis factor-alpha (TNF-α), interleukins 6 and 1β (IL-6, IL-1β), triggering interferon-α (IFN-α) production by plasmacytoid dendritic cells and subsequent IL-12/IL-23–mediated activation of T-helper cells (Kamata and Tada 2023; Man et al. 2023; Wang et al. 2024). These lymphocytes trigger IL-17 and IFN-γ–mediated inflammation and keratinocyte hyperproliferation. Ki-67, a well-known marker of cell proliferation, is notably elevated in psoriasis and is associated with keratinocyte overactivity as well as the severity of the disease (Jin et al. 2021; Zhang et al. 2023). Pro-inflammatory cytokines, including TNF-α and IL-23, enhance NF-κB transcriptional activity, resulting in elevated nuclear factor kappa B (NF-κB) levels in psoriatic skin (Chen et al. 2022; Zhang et al. 2023). Furthermore, these cytokines can activate the signal transducer and activator of transcription 3 (STAT-3), which promotes T cell differentiation, epidermal hyperplasia, and chronic inflammation (Chen et al. 2022; Li et al. 2023a, b).

Psoriasis is a chronic, incurable disease, and current treatments aim to improve skin appearance. Available therapies include topical agents, systemic drugs, and phototherapy; commonly used topicals such as vitamin D analogs and corticosteroids reduce inflammation, pruritus, and scaling (Kleyn et al. 2019; Koo et al. 2017). Systemic immunosuppressants improve psoriasis by modulating inflammation and keratinocyte growth (Lebwohl and Ali 2001). Moreover, biologic therapies targeting IL-17, IL-23, and TNF-α effectively modulate key pathogenic pathways and limit disease progression in psoriasis (Radi et al. 2020; Yost and Gudjonsson 2009). Phototherapy is often recommended for better treatment outcomes (Kemény et al. 2019). Despite their efficacy, current therapies may cause adverse effects, including skin atrophy and increased infection risk with long-term corticosteroid use (Yasir et al. 2025). Additionally, prolonged low-dose immunosuppressant therapy can result in liver and gastric toxicity, myelosuppression, and hair loss (Kremer 2024).

This data underscores the need for alternative psoriasis therapies with fewer side effects. Developing novel topical formulations can improve drug delivery to lesions while minimizing systemic exposure, thereby enhancing patient safety and adherence (Sindrilaru et al. 2020). Nanoparticle technologies offer a promising approach for developing safe, effective, and disease-specific topical therapies. Compared to conventional treatments, nanoparticles enhance drug delivery to target cells, improve therapeutic outcomes, reduce side effects, protect against degradation, increase stability and solubility, including for hydrophobic compounds, and enable controlled release at desired skin concentrations (Palmer and DeLouise 2016; Wollina et al. 2019). Nanoparticle delivery can enhance efficacy, safety, and durability in psoriasis therapy (Sindrilaru et al. 2020). Nano-drug delivery systems are broadly classified into metal-, polymer-, and lipid-based nanocarriers. Lipid-based carriers improve adhesion to the skin surface, prolong contact with the stratum corneum, and enhance penetration of active compounds. Nanoemulsions (NEs), formed by dispersing immiscible water and oil phases, typically have droplet sizes of 20–500 nm (Zhang et al. 2022). NEs deliver drugs evenly across the skin and are composed of aqueous and oil phases with surfactants, forming stable, transparent droplets ≤ 200 nm (Faria-Silva et al. 2020). NEs enable effective skin delivery with minimal irritation and high drug-loading potential (Salim et al. 2016). Incorporating NEs into creams enhances skin adherence, drug penetration, and ease of application. The resulting nanocream (NC) maximizes NE efficacy and improves patient compliance (Rai et al. 2018).

Natural products offer a safer alternative to synthetic drugs in psoriasis management (Sindrilaru et al. 2020). Medicinal plants offer structurally diverse compounds with broad therapeutic potential (Fuentes-Duculan et al. 2010). Polyphenols, present in fruits, vegetables, and herbs, are phenol-rich compounds with antioxidant and anti-inflammatory effects and diverse pharmacological function. (de Lima Cherubim et al. 2020; Fejér et al. 2019; Singla et al. 2019). Polyphenols positively affect psoriasis via antioxidant and anti-inflammatory actions (Faria-Silva et al. 2020). Based on their biological activities, the polyphenols in the aerial parts of *Deverra tortuosa* (*D*. *tortuosa)* and *Deverra triradiata* (*D*. *triradiata)* have been demonstrated as potential sources for treating psoriasis.

The Apiaceae family includes economically important aromatic plants used as food, spices, condiments, ornamentals, and in traditional medicine. They also have applications in the food, pharmaceutical, and cosmeceutical industries (Önder et al. 2020; Shelef 2003). Apiaceae species are widely employed in traditional medicine (Li et al. 2023a, b). Several Apiaceae species are rich in bioactive compounds with antioxidant, antimicrobial, anti-inflammatory, and diverse health-promoting effects, including antidiabetic and cardioprotective activities (Belbachir et al. 2024; Derouich et al. 2020; Li et al. 2023a, b). The genus Deverra DC. (Syn Pituranthos Viv) has nine species and four subspecies; *D. tortuosa* and *D. triradiata* are common in South Sinai, Egypt (Täckholm 1974). The Deverra genus is traditionally used in Sinai Bedouin medicine as a carminative, antiasthmatic, and for parasites (Elmosallamy et al. 2021). *D. tortuosa,* a desert shrub with edible fragrance, is found in sandy areas of the Arabian share-region, including Egypt (Boulos 2000). Its uses include analgesic, carminative, antiasthmatic, diuretic, and treatments for stomach pain, parasites, rheumatism, fever, diabetes, hepatitis, hypertension, and menstrual regulation (Mahran et al. 1989; Vérité et al. 2004). Phenolic compounds and some flavonoids were identified in aerial parts (Ahmed et al. 2021; Alhumaydhi et al. 2021; Selim et al. 2020); moreover, some flavonoids were isolated from its aerial parts (Mostafa et al. 2020; Singab et al. 1998). Additionally, furano- and dihydrofuranocoumarins were discovered in the non- polar fractions of *D*. *tortuosa* seeds (Oueslati et al. 2021) and roots (Abdel-Kader 2003; Halim et al. 1989). From a biological perspective, the seeds demonstrated antibacterial and cytotoxic activity (Oueslati et al. 2021), while the aqueous alcoholic extract of the aerial parts showed antioxidant, antimicrobial, antitumor (Selim et al. 2020; Singab et al. 1998), hematological, and antidiabetic activities (Oueslati et al. 2021). *D. triradiata,* a smooth, leafless desert shrub along the Mediterranean and South Sinai, has traditional uses similar to *D. tortuosa* (Halim et al. 1989). Few reports exist on phenolic compounds and coumarins in the aerial parts and roots (Elmosallamy et al. 2021) and on coumarins in the roots (Al-Meshal et al. 1983; Halim et al. 1989) and shoot extracts (Ashkenazy et al. 1983; Halim et al. 1989). Additionally, cytotoxic (Al-Meshal et al. 1983; Ashkenazy et al. 1983; Elmosallamy et al. 2021) and antioxidant activities have been evaluated (Elmosallamy et al. 2021). Continuing our research on the genus Deverra (Kamel et al. 2022), this study aims to analyze the phenolic profile of the defatted 80% aqueous ethanol extract (DAEE) from the aerial parts of *D*. *tortuosa* and *D*. *triradiata*, and to assess the in vivo anti- psoriatic effects of the developed NC.

## Materials and methods

### Plant material

Aerial parts of *D. tortuosa (*Desf.) DC and *D. triradiata* Hochst. Ex-Bioss were collected in February 2021 from the Wadi Degla protectorate in Cairo, Egypt. These species were identified by Prof. Dr. Abduo Marie Hamed, a Plant Ecology expert et al.-Azhar University's Faculty of Science, Nasr City. A voucher specimen (01 DTO 2021 and 02 DTI 2021) is kept in the Pharmacognosy Department at the Faculty of Pharmacy, Al-Azhar University for Girls.

### General chemicals

Ferric chloride, *Naturstoff* reagent, and MeOH for HPLC, Folin-Ciocalteu reagent, gallic acid (Sigma-Aldrich, Germany); formic acid (99%; Merck, Darmstadt, Germany). Standers of Kaempferol and quercetin-3-*O*-glucoside (GmbH & Co. KG, Vestenbergsgreuth, Germany by PhytoLab) while the remaining phenolics standards and, the Milli-Q SP reagent water system (Millipore – MA- USA) was used for water deionization to produce ultrapure water (resistivity of > 18 M cm); polyamide filter (0.2 m, Sartorius Stedim, Germany) was used for filtration of all HPLC solutions. Oleic acid and polysorbate 80 (Al-Nasr for chemicals, Abou-Zabal, Cairo, Egypt), propanol alcohol (Al-Goumhoria company for chemicals, Garden City, Cairo, Egypt), Jojoba oil, stearic acid, propylene glycol, white soft paraffin, and paraffin oil (Chemises company, New Borg El-Arab City, Egypt), Whatmann No.1 for paper chromatography (PC) (Whatmann Ldt, Maidstone, Kent, England). A topical cream with 5% imiquimod (IMQU) and tacrolimus, a topical corticosteroid, was supplied from a local pharmacy.

All other solvents used are of analytical grade (El Nasr Pharmaceutical CH. Co, Egypt).

### Preparation of the extract

Aerial parts air-dried powder of *D. tortuosa* (1.5 kg) and *D. triradiata* (1.15 kg) was extracted with 80% aqueous ethanol under reflux (5.0 L × 4). The solvents were evaporated under vacuum at low temperature, yielding 200 and 150 g of dry extract for the two species, respectively. The two extracts were defatted under reflux using petroleum ether (40–60 °C, 3 L × 3), followed by solvent evaporation to yield a dry defatted aqueous ethanol extract (DAEE), 145.8 g for *D. tortuosa* and 102.5 g for *D*. *tortuosa,* respectively. Further purification of the extracts was achieved by precipitation using an ethanol–water mixture (1:10), followed by filtration to remove the precipitated salts and sugars. The filtrate was evaporated to yield dry extract of nearly 123.6 and 82.4 g for *D. tortuosa* and *D. triradiata,* respectively. For the detection of phenolic compounds, the DAEE *was subjected to two-dimensional paper chromatography (2D-PCE) using n-butanol-AcOH-H*_*2*_*O (BAW, 4:1:5 v/v) and 15% AcOH for elution, followed by examination under UV light and spraying with FeCl3 and Naturstoff reagents.*

### Estimation of total phenolic content (TPC)

The estimation was performed using the Folin-Ciocalteu colorimetric method [Zilic et al. 2012; Salem et al., 20.25]. Absorbance was measured at 725 nm with a UV–visible spectrophotometer (Jasco V-730, Jasco Corporation, Japan). Results were derived from the calibration curve of the standard (gallic acid 50–300 μg/mL) using the following equation: y = 0.0237x + 0.0455 (R^2^ = 0.9976) (Figure S1).

The results were stated as gallic acid (mg) equivalent (mg GAE/mg) of dry weight.

### HPLC–MS/MS for phenolic content for aerial parts of *D. tortuosa* and *D. triradiata*

#### Preparation of stock solution

Stock solutions of DAEE and standards (500 mg/L) were prepared in methanol (HPLC) and stored at 5°C. Different concentrations were freshly prepared from it using MeOH (HPLC) and filtered through Phenex™ RC syringeless filters (4 mm × 0.2 μm, Phenomenex, Castel Maggiore, BO, Italy) before injection.

#### Condition for HPLC–MS/MS

The phenolic content of *D*. *tortuosa* and *D*. *triradiata* aerial parts was quantified using the modified method reported by Mustafa et al. (2022) and El-Deeb et al. (2024). Quantification of the phenolic content was performed using a modified version previously described (24. An Agilent 1290 Infinity series with a triple quadrupole 6420 HPLC-MS/MS (Agilent Technology, Santa Clara, California, United States), coupled with an ESI source, was used. The instrument operated in -ve and +ve ionization modes. Flow injection analysis (FIA) was used to optimize the MS/MS parameters for standards. Phenolic components were separated by injecting the diluted samples (1:5) into the Phenomenex Synergi Polar-RP C18 column (250 x 4.6 mm, 4 µm). The mobile system is water and MeOH for solvent A and B respectively, each one containing formic acid (0.1 %) as well as, its preparation being isocratic mode, 20 % B (0 −0.1 min); gradient mode, 20 - 85 % B (1–25 min); isocratic mode, 85% B (25–26 min); gradient mode, 85-20% B (26–32 min), The column and the drying gas temperature in the ionization source were adjusted at 30 and 350 °C respectively. The flow rate of the gas and injected volume (2 μL) were 12 L and 0.8 mL min-1, respectively, and the capillary voltage and nebulizer pressure were 4000 V and 55 psi, respectively. For quantitation, the peak area was integrated after recognition in dynamic-multiple reaction monitoring (dynamic-MRM) mode, and the maximum production was used; for qualitative analysis, the remaining ions were used. The retention time of each compound was established at 2 min.

### Development of pseudo-ternary phase diagram for both extracts

Our development was based on previous work, with detailed methods provided in the supplementary data (Kamel et al. 2022). Briefly, the oily phase consisted of a 1:1 weight ratio of oleic acid and jojoba oil, while polysorbate 80 combined with propanol acted as the surfactant/cosurfactant. These components were mixed at various ratios **(**Table S1**)** and combined with the oily phase. Deionized water was gradually added until the mixture reached a stable turbidity. All weights were recorded and expressed as weight-to-weight percentages (w/w%). A pseudo-ternary phase diagram was constructed using Chemix School's ternary diagram software.

### Preparation of a nanoemulsion (NE) from the two extracts

Based on the constructed pseudo-ternary phase diagram, specific concentrations of the oil mixture, the surfactant/cosurfactant (SAA/Co-SAA) mixture, and water were selected to generate the subsequent NEs. Various NE formulas were developed using the plant extract within the aqueous phase. Three distinct NE formulations were developed using a spontaneous emulsification method (Aswathanarayan and Vittal 2019) with minor adjustments. The blank formulation (F1) was prepared by mixing oleic acid, jojoba oil, Tween 80, and propanol using a hot plate stirrer (Wisestir MSH-20D, Belgium) to create the unloaded blank cream (F1). Furthermore, the loaded creams were prepared by dispersing the NE (8% plant extracts) in the aqueous phase with stirring for 10 min, then mixing it with the oil phase under constant stirring until the mixture reached room temperature. Two formulations (F2 and F3) were prepared from similar concentrations of aqueous and oily phases in the presence of different NE loaded with two plant extracts (*D. tortuosa* and *D. triradiata*), as shown in Table S2.

### Preparation of the cream formulation

Topical NC was formulated from the previously prepared NE. The aqueous and oily phases of the cream were designated to be compatible with the NE contents, and their ratio was determined to prepare an o/w semisolid emulsion (vanishing cream) according to (Iskandar and Silalahi 2016) with some modifications. The oily phase was created by melting white soft petrolatum, liquid paraffin, and stearic acid at approximately 60–70 ºC for 20 minutes, while the aqueous one was prepared by mixing propylene glycol, polysorbate 80, and purified water on a hot plate stirrer at 50 ºC. Furthermore, the NE (8% of plant extracts) was spread in the aqueous phase with stirring (10 minutes), then mixed with the oil phase using continuous stirring till the cream cooled to room temperature. Twenty-five grams of plant extract were incorporated into each 100 gm of the NC, resulting in a final plant extract concentration of 2% (w/w) of plant extract in NC. Three formulations (F1, F2, and F3) were prepared from different concentrations of aqueous and oily phases.

### Physicochemical evaluation of NE

#### Determination of NE globule size and Zeta potential

The average droplet diameter, size distribution, and surface zeta potential were measured for both the plain formulas and the two NE. For each formula, 100 µL was diluted with deionized water and analyzed to determine these parameters using dynamic light scattering (DLS; Beckman Coulter Delsa nanoparticle size analyzer, USA) at 25 °C and a 160° angle. All measurements were performed in triplicate.

#### Visual examination for the prepared NC

NCs were visually inspected for color, appearance, uniformity, and consistency (Ugandar and Deivi 2019).

#### pH determination for the prepared NC

A pH meter (Jenco large pH/mV/Temperature Meter Kit—6173KB, USA). was used to measure the pH values of the plain cream and the extracts loaded NC. Briefly, 5 g of the cream was dispersed in 95 mL of water, then the electrode was immersed in the suspension, and the reading was recorded. The reading was recorded in triplicate (Kuntal Das et al. 2012).

#### Rheological properties for the prepared NC

The viscosity of the creams (F1, F2, F3) was determined using a Brookfield-B-One Plus viscometer (Chennai, Tamil Nadu, India) equipped with a spindle at 25 ºC by setting 500 mm stainless steel rod spindle at a shear rate of 50 rpm, and the readings were taken for 2 min. The results were carried out in triplicate. The viscosities were compared by one-way analysis of variance (ANOVA) at p < 0.05.

#### Effect of storage for the prepared NC

The creams (F1, F2, F3) were stored at different temperatures (25 ± 1 ºC and 40 ± 1 ºC) and relative humidity (75 ± 5%) to simulate the storage phenomena under different conditions, then the stability was evaluated after 3 and 6 months through which the cream was evaluated for physical changes (coalesce, cracking or phase separation), pH value, and rheological properties (Kuchekar and Bhise 2012; Matangi et al. 2014).

### In vivoanti-psoriatic activity

#### Experimental animals

Thirty Male BALB/c mice, 6–8 weeks old (20–25 g), were obtained from the Egyptian Organization of Biological Products and Vaccines (Helwan, Egypt). One week prior to the study, the mice were acclimated to the appropriate conditions (temperature, 23 °C ± 2 °C; 12:12 dark–light cycle), with free access to food and water. All animal experiments adhered to the guidelines of the Animal Care and Use Committee at the Faculty of Pharmacy, Al-Azhar University (Protocol #: AZU: 370–2023), and followed the European Community Directive (86/609/EEC), a national regulation on animal care that aligns with the NIH Guidelines for the Care and Use of Laboratory Animals (8th edition).

#### Experimental design

**Fig. 1:**
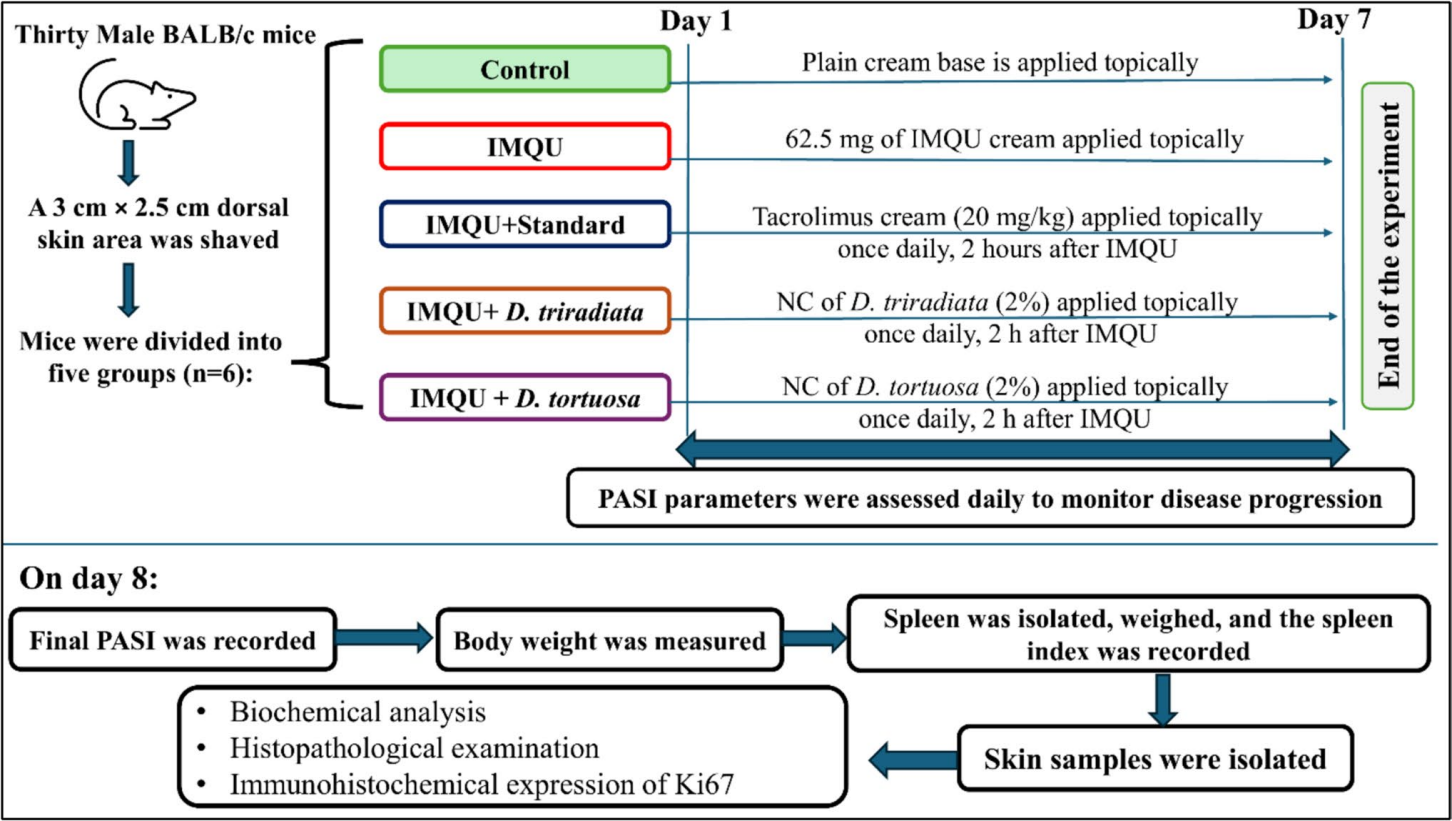
The experimental design of the study

The dorsal skin of the mice was shaved to create a hairless area of 3 cm × 2.5 cm, and then the mice were randomly assigned to five groups (n = 6). The control group received the plain cream base topically. The IMQU group received 62.5 mg of IMQU cream topically applied for seven days (Chen et al. 2022) while the standard group was treated topically with tacrolimus cream (20 mg/kg) 2 h after IMQU cream application (Gangadevi et al. 2021). IMQU + *D. triradiata* NC group, mice topically received NC of *D. triradiata* (2%) once daily, 2 h after IMQU application. IMQU + *D. tortuosa* NC group, mice topically received NC of *D. tortuosa* (2%) once daily, 2 h after IMQU application. All treatments were applied once daily for seven consecutive days. On day 8, mice were evaluated for psoriasis area and severity index (PASI) and then sacrificed. The experimental design is illustrated in Fig. 1.

#### Determination of the body weight

Throughout the experiment, it was measured daily for each mouse.

#### Psoriasis area and severity index (PASI)

Erythema, scaling, and thickness were scored separately using an objective scoring system: none: [0], slight: [1], moderate: [2], marked: [3], very marked: [4] (Guo et al. 2022). During the experiment, PASI parameters (erythema, scaling, and thickness) were assessed daily to monitor disease progression. PASI on day 8 is considered an endpoint assessment. After assessing PASI on day 8, mice were anesthetized using thiopental sodium (40 mg/kg/i.p). (Soliman et al. 2023)**,** and sacrificed by cervical dislocation**.** Skin lesions and spleens were harvested. Formal saline (10%) was used to fix some of the skin lesions for histopathological and immunohistochemical investigations, while a phosphate buffer saline was used to homogenate the others’ skin lesions for biochemical parameters.

#### Spleen weight and spleen to body weight index

Spleen weight was recorded for each mouse immediately after sacrifice. Body weight was measured daily throughout the experiment, and the final body weight recorded on day 8, immediately before sacrifice, was used to calculate the spleen-to-body weight index for each mouse using the following formula:

$$Spleen\; index =\frac{\text{Spleen weight }(\mathrm{g})}{\text{Body weight on day }8 (\mathrm{g})}$$

#### Biochemical parameters

*Assessment of TNF-α, IL-6, IL-17 and NF-κB Levels with ELISA technique:* ELISA kits for TNF-α (Cat No: 430904, Biolegend, USA), IL-6 (Cat No: SEA079Mu, Cloud-Clone Corp, USA), IL-17 (Cat No: SEA063Mu, Cloud-Clone Corp, USA), and NF-κB (Cat No: EM1230, Fine Test, China) were used according to the manufacturer’s instructions. Skin lesions collected from each mouse were homogenized in phosphate-buffered saline (PBS) and centrifuged at 10,000 × g for 10 min at 4 °C to obtain the supernatant, which was used for ELISA measurements. All assays were conducted according to the manufacturer’s instructions. Optical density was measured using a microplate reader at the recommended wavelength. Cytokine levels were expressed as pg/mg of tissue protein, and NF-κB as ng/mg of tissue protein.

##### Determination of STAT3 by Western blot method

Ready PrepTM protein extraction kit (Cat No: 163–2086, Bio-Rad Inc., USA) was used to extract total skin tissue proteins, and Bradford Protein Assay Kit (Cat No: SK3041, Bio Basic Inc., Canada) was used for protein quantification. For separation and denaturation of proteins, Sodium Dodecyl Sulfate Polyacrylamide Gel Electrophoresis (SDS-PAGE) (Cat No: 161–0181, Bio-Rad Laboratories Inc., USA) was employed. After protein separation, samples were incubated with primary antibody against STAT3 (1:1000) (Cat No: ab68153, Cambridge, UK) at 4 °C overnight. The samples were rinsed with tris-buffered saline and Tween 20, and then the membranes were incubated with goat anti-rabbit HRP-conjugated secondary antibody at 25 °C, followed by washing in buffer. Clarity TM Western ECL substrate (Bio-Rad, USA) was applied as a chemiluminescent substrate, and the protein bands were visualized using a CCD camera-based imager. Chemi Doc MP Imager was used to analyze the images with beta-actin (β-actin) serving as the control sample.

### Histopathological Investigations

For three days, skin samples were fixed in 10% neutral buffered formalin. They were then dehydrated through a series of ethanol concentrations, cleared in xylene and embedded in a paraffin embedding medium (Paraplast®). The samples were sectioned into 5 μm thickness using a rotary microtome and then stained with hematoxylin and eosin (H&E) for microscopic investigations. All protocols were carried out according to (Culling 2013).

### Immunohistochemical analysis for Ki67

Skin tissue sections (five micrometers) were deparaffinized and treated with hydrogen peroxide (0.3%) for twenty minutes. Phosphate-buffered saline (PBS) was used for sample washing before they were incubated overnight at 4 °C with anti-ki-67 (1:200 dilutions) as the primary antibody (Cat No: GTX16667, GeneTex Co., USA). Samples were washed and incubated with a secondary antibody using the HRP Envision kit (DAKO) for twenty minutes, followed by a 15-min incubation with diaminobenzidine after washing. Afterwards, the samples were stained with hematoxylin as a counterstain, cleared with xylene, and then coverslipped for microscopic examination.

### Morphometric and immunohistochemical analysis

For each sample, six distinct fields were analyzed to determine the average epidermal thickness in H&E-stained skin tissue sections. Ki-67 expression was also evaluated using immunohistochemistry. The samples were imaged and processed with the Leica application module for histological analysis (Leica Microsystems GmbH, Germany).

### Statistical analysis

All data are presented as mean ± standard error of the mean (SEM). Statistical analyses were performed using GraphPad Prism version 8. One-way analysis of variance (ANOVA) was used to compare differences among multiple groups, followed by Tukey’s post-hoc test for pairwise comparisons between all experimental groups. Differences were considered statistically significant at p < 0.05. For all comparisons, the control group and the IMQU-treated group were included as references to evaluate the effects of standard treatment and nanocream formulations. Graphs were plotted to visualize group differences, and all statistical details are reported in the figure legends.

## Results

### Chromatographic investigation of the extract

The *2D-PC for the DAEE revealed a complex mixture of phenolic compounds under UV light (254 and 360 nm). It also gave a different color under UV light after spraying the chromatogram with Naturstoff reagents. Therefore, the qualitative and quantitative polyphenolic profiles of the DAEEs of the two Deverra species were evaluated using HPLC–MS/MS. Thirty-eight* phenolic *standards were used, and the analysis was done* according to the acquisition parameters epitomized in Table S3.

### Quantification of phenolic contents from two Deverra species

The TPC of the two Deverra species was measured using a colorimetric assay. The results showed that the TPC was similar across the two species, with values of 34.867 ± 0.66 and 34.319 ± 0.41mg GAE/g for *D. tortuosa* and *D. triradiata*, respectively. Previously, the TPC of the *D. tortuosa* shoots and aerial parts methanol extracts was reported as 109.00 ± 3.59 and 7.81 mg GAE/g, respectively (El-Amier et al. 2024; Ahmed et al. 2021). whereas that of the methanol extract of *D*. *triradiata* aerial parts becomes 67.8 ± 0.39 mg tannin acid/g (Elmosallamy et al. 2021). Differences between our data and previous reports could stem from variations in collection sites and extraction conditions, including solvent type, extraction duration, and temperature (Moharram et al. 2025).

### HPLC–MS/MS investigation for the extracts of the two species

A total of twenty-three and twenty-two compounds were identified in the aerial portions of *D. tortuosa* and *D. triradiata*, respectively. The total phenolic content was assessed by calculating the average content (RSD% ≤ 5.3) of each identified phenolic. The findings indicated that the total phenolic content in *D. triradiata's* aerial parts surpasses that of *D. tortuosa*, measuring 37,434.853 and 16,701.323 μg/kg, respectively (Tables 1 and 2). Additionally, the phenolic acid content in *D. triradiata* (27,722.096 μg/kg) is notably higher than in *D. tortuosa* (4236.689 μg/kg), while the flavonoid content in *D. tortuosa* (12,455.133 μg/kg) exceeds that in *D. triradiata* (9045.934 μg/kg). In both species, vanillic acid is the predominant phenolic acid, recorded at 2565.610 μg/kg in *D. tortuosa* and 22,684.270 μg/kg in *D. triradiata.* Furthermore, both chlorogenic acid and p-hydroxybenzoic acid are abundant in *D. triradiata*. Rutin represents the primary flavonol in *D. triradiata* (7827.987 μg/kg), while hyperoside (5002.950 μg/kg) and rutin (2348.645 μg/kg) are the principal flavonols found in *D. tortuosa*. Furthermore, flavan-3-ols, dihydrochalcones, and *trans*-cinnamic acid were identified in trace levels in both species, while flavanones were only present in trace amounts in *D. triradiata*. Additionally, anthocyanins and stilbenes were not detected in either species.

**Table 1:** Phenolic compounds identified in DAEE of *D. tortuosa* and *D. triradiata* aerial parts by HPLC–MS/MS (RSD ≤  ± 7.01)

No	Compound name	*D. tortuosa*	*D. triradiata*
	**Phenolic acids**		
1	Gallic acid	336.104	162.645
2	Neochlorogenic acid	1.917	34.665
3	Chlorogenic acid	157.647	**1958.498**
4	*p*-Hydroxybenzoic acid	160.925	**1199.761**
5	3-Hydroxybenzoic acid	n.d	30.289
6	Caffeic acid	96.727	131.197
7	Vanillic acid	**2565.610**	**22,684.270**
8	Syringic acid	335.851	771.474
9	p-Coumaric acid	178.337	565.990
10	Ferulic acid	47.054	55.890
11	3.5-Dicaffeoylquinic acid	2.612	127.417
12	Ellagic acid	353.905	n.d
	**Flavonoids**		
	**A) Anthocyanins**		
13	Delphinidin 3.5 diglucoside	n.d	n.d
14	Delphinidin3-galactoside	n.d	n.d
15	Cyanidin-3-glucoside	n.d	n.d
16	Petunidin-3-glucoside	n.d	n.d
17	Pelargonidin-3-rutinoside	n.d	n.d
18	Pelargonidin-3-glucoside	n.d	n.d
19	Malvidin-3-galactoside	n.d	n.d
	**B) Flavonols**		
20	Rutin	**2348.645**	**7827.987**
21	Isoquercitrin	4142.183	480.743
22	Quercitrin	4.459	2.300
23	Myricetin	n.d	n.d
24	Kaempferol-3-glucoside	23.820	12.267
25	Quercetin	215.798	67.575
26	Isorhamnetin	652.451	n.d
27	Hyperoside	**5002.950**	655.062
28	Kaempferol	64.827	n.d
	**C) Flavan-3-ols**		
29	Catechin	n.d	n.d
30	Epicatechin	1.351	n.d
31	Procyanidin B2	n.d	47.032
32	Procyanidin A2	7.181	23.802
	**D) Dihydrochalcones**		
No	**Compound name**	***D. tortuosa***	***D. triradiata***
33	Phloridzin	0.969	0.282
34	Phloretin	n.d	n.d
	**E) Flavanones**		
35	Hesperidin	n.d	595.707
36	Naringin	n.d	n.d
	**Stilbenes**		
37	Resveratrol	n.d	n.d
	**Non-Phenolic acids**		
38	*Trans*-cinnamic acid	75.819	476.165

n.d.: not detected

**Table 2:** Quantitative estimation (μg/Kg) of various phenolic classes identified in *D. tortuosa* and *D. triradiata* using HPLC–MS/MS

Content		
	*D.tortuosa*	*D. triradiata*
Anthocyanins	**-**	**-**
Flavonols	**12,455.133**	**9045.934**
Flavan-3-ols	8.532	70.834
Dihydro chalcones	0.969	0.282
Flavanone	**-**	595.707
Phenolic acids	**4236.689**	**27,722.096**
Total phenolic content	**16,701.323**	**37,434.853**

#### Phenolic acids and/derivatives

Based on comparisons with authentic samples, deprotonated or protonated molecular ion peaks, and fragment ions, eleven phenolic acids were tentatively identified in both *D*. *tortuosa* (**1–4** and **6–12**) and *D*. *triradiata* (**1**–**11**), as shown in Table 1. According to the standard classification of phenolic acids, compounds **1**, **4**, **5**, **7**, and **8** were grouped as hydroxybenzoic acid derivatives, while compounds **2**, **3**, **6**, **9**, **10**, and **11** were categorized as hydroxycinnamic acids; compound **12** was considered a polyphenolic acid analog (Xie et al. 2024). Compound 1 was identified in negative-ion mode as gallic acid (*m/z* 169.0 [M-H]^−^). Metabolites **4** and **5** were identified as *p*-hydroxybenzoic acid and 3-hydroxybenzoic acid, both with [M-H]^−^ at *m/z* 137 but different fragment ions (Table S2). Additionally, compounds **2** and **3** were identified as neochlorogenic acid and chlorogenic acid, both with [M-H]^−^ = 353 but different fragment ions. Compounds **6** and **11** were identified as caffeic acid ([M-H]^−^ 179.0) and its derivative, 3,5-dicaffeoylquinic acid ([M-H]^−^ 514.9). Metabolites **7** and **8** were labeled as vanillic acid ([M-H]^−^ 176.0) and syringic acid ([M-H]^−^ 196.9). Meanwhile, compounds **9**, **10**, and **12** were identified as *p*-coumaric acid ([M-H]^−^ 163.0), ferulic acid ([M-H]^−^ 193.0), and ellagic acid ([M-H]^−^ 465.2224), respectively. It was reported that compounds **1**, **3**, **6**, **8** -**10**, and **12** were previously reported from the aerial parts of *D*. *tortuosa* (Benabderrahim et al. 2019; Selim et al. 2020). Compounds **1**, **1**, **3**, and **12** were also identified in the aerial parts of *D*. *triradiata* (El Mesallamy et al. 2021), while the other compounds were identified in both species for the first time.

#### Non-phenolic acid

*Trans*-cinnamic acid is identified in the aerial parts of both species with *m/z*M + H]^+^ at *m/z* 149. This compound was detected only from the *D*. *tortuosa* aerial part (Selim et al. 2020).

#### Flavonoids

Flavonoids have a fifteen-carbon skeleton with three rings: ring A (6 carbons), ring C (3 carbons), and ring B (6 carbons). They are classified into various subclasses based on their core structure, unsaturation pattern, and oxidation level (do Nascimento et al. 2022; Moharram et al. 2025). Specifically, flavonoids are divided into nine groups, including flavonols (**20**–**28**), flavan-3-ols (**30**–**32**), and dihydrochalcones (**33**), as shown in Table 1. Compounds **20**, **21, 22**, **25**, **26**, and **27** belong to quercetin aglycone with a fragment at *m/z* 301.0 in negative mode or 303 in positive mode. The four compounds were identified as rutin **20** ([M-H]^−^ 609.0), isoquercitrin **21** ([M-H]^−^ 463.0), quercitrin **22** ([M-H]^−^ 446.0), quercetin **25** ([M-H]^−^ 300.99), isorhamnetin **26** ([M-H]^−^ 314.99), and hyperoside **27** ([M + H]^+^ 465.01). Additionally, compounds **24** and **28** were identified as kaempferol-3-glucoside ([M-H]- 447.0) and kaempferol **27** ([M + H]^+^ 287.01), respectively. All compounds were found in the flowers and roots of both species. Compounds **20**–**22**, **24**–**26**, and **28** were previously reported from the aerial parts of *D*. *tortuosa* (Benabderrahim et al. 2019; Selim et al. 2020; Alhumaydhi et al. 2021). While compounds **20** and **25** were reported from the aerial parts of *D*. *triradiata* (El Mesallamy et al. 2021). The other compounds were identified in both species for the first time.

#### Flavan-3-ols

Epicatechin **30** ([M-H]^−^ 299.00) and procyanidin A2 **32** ([M-H]^−^ 575.0) were identified in the *D*. *tortuosa*, while procyanidin B2 **31** ([M-H]^−^ 576.99) and procyanidin A2 **32** were identified in *D*. *triradiata* were identified in *D*. *triradiata.* Compound **30** was detected only *in the aerial part of D. tortuosa* (Benabderrahim et al. 2019). The other compounds were identified in both species for the first time.

#### Dihydrochalcones

Phloridzin **33** ([M-H]^−^ 435.39) was identified in both species for the first time.

#### Flavanones

Hesperidin **35** ([M + H]^+^ 611.01) is the only flavanone identified in the *D*. *triradiata* aerial part, and it was identified in both species for the first time.

### Preparation of nanoemulsion from the aerial parts extract of two species

#### Development of pseudo-ternary phase diagram for both extracts

It was formulated with an oleic/jojoba mixture, Tween 80/propanol, and water for NE development (Kamel et al. 2022). A certain point was selected in the white area of the ternary phase (one phase) that was formulated with 6.3w/w % of oleic and jojoba oil as oily phase, 31.3 w/w% of tween 80 & propyl alcohol as SAA/CO-SAA mixture, and 62.4 w/w% of water for the preparation of the plain formula (F1). The plant extract was added to the aqueous phase of the NE at a concentration of 8 w/w% to prepare the *D. tortuosa* (F2) and *D. triradiata* (F3) formulae. Furthermore, the prepared NE-loaded plant extracts were incorporated into the aqueous phase for the formulation of a topical vanishing cream, as mentioned in the experimental part.

#### Droplet size, polydispersity index, and zeta Potential of the NEs

**Fig. 2:**
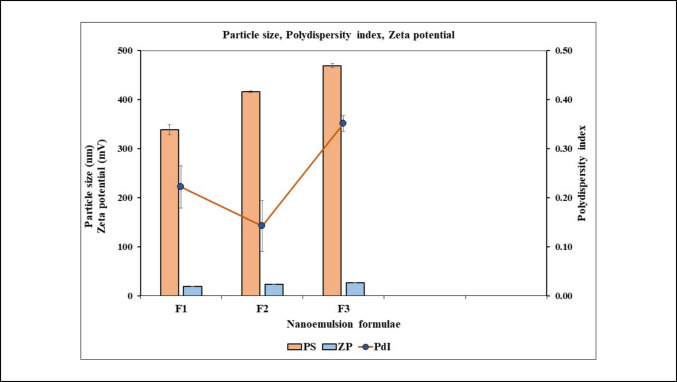
Particle size, zeta potential, and polydispersity index of the formulations

The droplet sizes for the blank and extract-loaded NE formulas range from 338.5 ± 10.2 to 469.2 ± 4.2 nm (Table 3 and Fig. 2). The extract-loaded formulas have larger globule sizes than the blank due to the presence of extracts, which alter the aqueous phase and increase droplet size. The polydispersity index (Table 3; Fig. 2) did not exceed 0.35 ± 0.01, indicating a narrow particle size distribution and uniform droplets, which improve topical application. Zeta potential measurements showed surface charges between 18.8 ± 0.22 mV and 26.2 ± 0.74 mV (Table 3 and Fig. 2). These results align with previous data, where particles with surface charges between − 10 and + 10 mV are neutral, and those with charges ranging from −30 mV to + 30 mV are strongly anionic or cationic, thereby enhancing sample stability.

**Table 3:** Droplet Size, Polydispersity Index, and Zeta Potential of the NEs

Formula	PS (nm)	ZP (mv) positive	PDI	pH value
F1 (Plain cream)	338.50 ± 10.2	18.80 ± 0.22	0.22	6.9 ± 0.37
F2 (Cream with *D. tortuosa* extract)	416.10 ± 1.3	23.50 ± 0.45	0.14	6.5 ± 0.29
F3 (Cream with *D. triradiata* extract)	469.20 ± 4.2	26.20 ± 0.74	0.35	6.6 ± 0.11

#### Visual examination for the prepared NC

The prepared NC formulations exhibited a viscous, semi-solid, homogeneous appearance, a smooth texture, and an off-white to limeade color, with an acceptable odor.

#### pH determination for the prepared NC

**Fig. 3:**
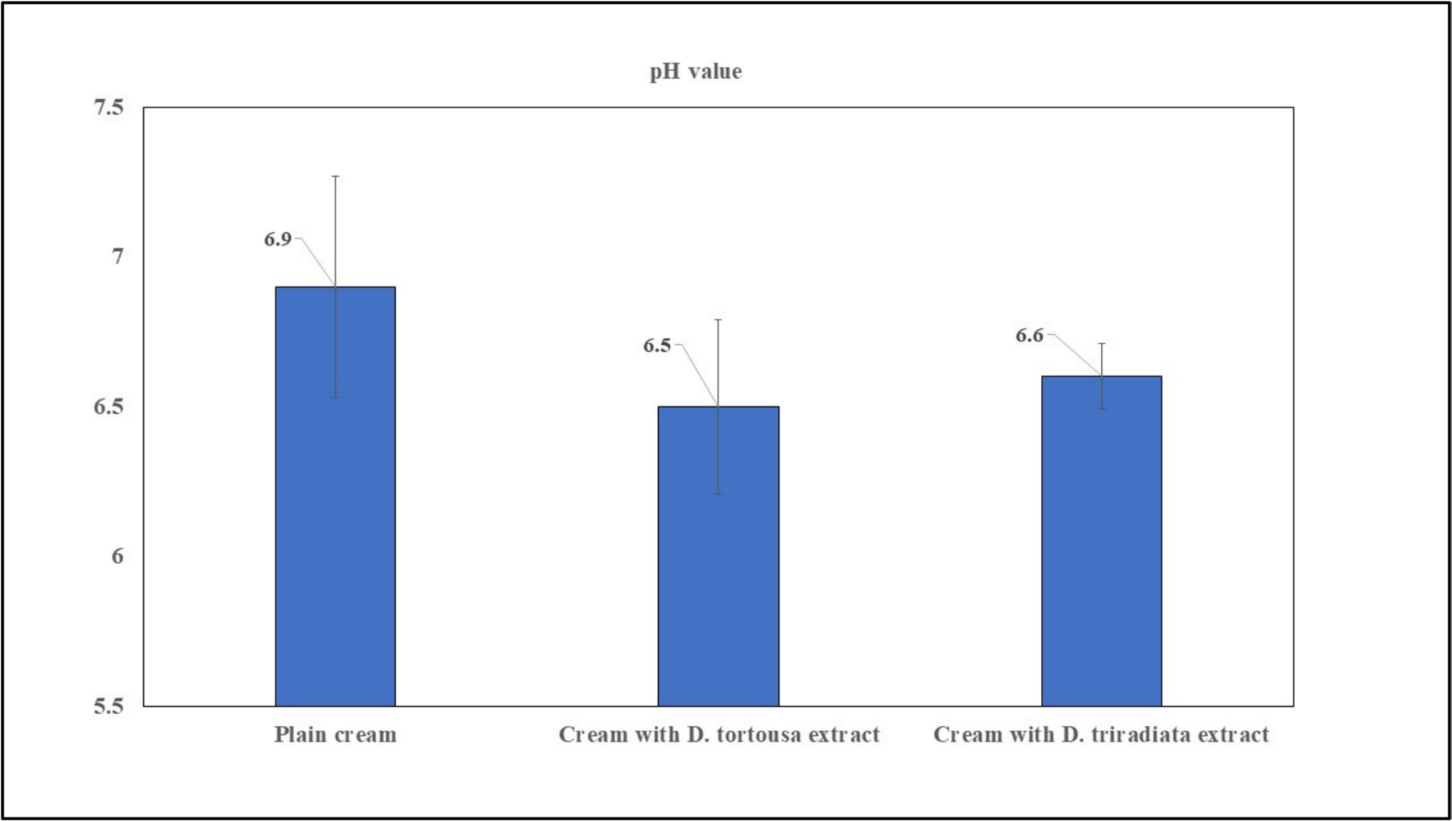
pH value of creams

The pH values of all samples range from 6.5 ± 0.29 to 6.9 ± 0.37 (Table 3, Fig. 3), which aligns with normal skin pH (4–6) to prevent skin irritation (Ali and Yosipovitch 2013).

#### Rheological properties for the prepared NC

The viscosity of the prepared NC was in the range of 3100–3500 cps. This proves that the cream was easily spreadable by a small amount of shear. The obtained results facilitate the application of the cream to the skin and do not require much rubbing during administration (Uddin et al. 2019)**.**

#### Effect of storage for the prepared NC

The reported results of the NC evaluation exhibited no significant change upon storage, which confirms the high stability of the prepared formulae (Table 4).

**Table 4:** Effect of storage for the prepared NC

^°^C	Evaluation	F1 (0 M)	F1 (3 M)	F1 (6 M)		F2 (0 M)	F2 (3 M)		F2 (6 M)	F3 (0 M)	F3 (3 M)	F3 (6 M)
25°C	Appearance	Homogenous				Homogenous				Homogenous		
	Texture	Smooth				Smooth				Smooth		
	Color	Off-white				Beige-Limeade				Beige-Limaded		
	pH	6.9	6.9		6.8	6.5	6.8	6.5		6.6	6.7	6.7
	Rheology (cP)	3180	3170		3170	3350	3380	3400		3480	3510	3495
40 ^°^C	Appearance	Homogenous				Homogenous				Homogenous		
	Texture	Smooth (slightly soft)				Smooth(slightly soft)				Smooth (slightly soft)		
	Color	Off-white				Beige-Limeade				Beige-Limeade		
	pH	6.9	6.8		6.7	6.5	6.5	6.8		6.6	6.7	6.7
	Rheology (cP)	3180	3150		3100	3350	3300	3250		3480	3440	3400

### In vivo anti-psoriatic activity

#### NC ameliorated PASI in IMQU-induced psoriatic lesions in mice

**Fig. 4:**
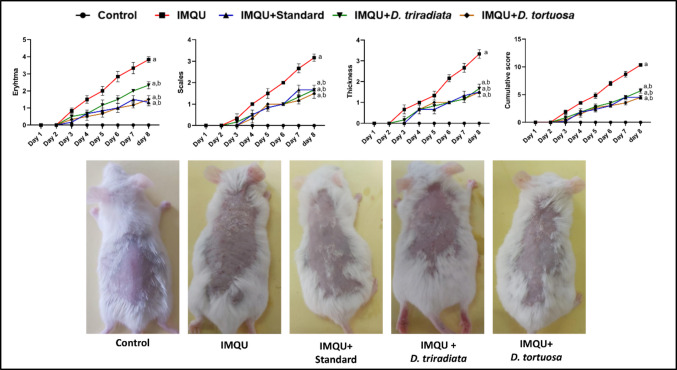
***D. tortuosa* and *D. triradiata* NC decreased PASI in IMQU-induced psoriasis in mice.** Data expressed as M ± SE, (n = 6). The test for significance was performed on day 8 using ANOVA test followed by Tukey’s post-hoc test (*p* < 0.05). The significance was expressed using the following symbols: a: significant from control, b: significant from IMQU

Applying IMQU topically promotes the formation of psoriatic skin lesions in mice, as evidenced by a significant (*p* < 0.05) 10.3-fold increase in PASI scores compared to healthy mice. On the other hand, NC of *D. triradiate and D. tortuosa,* as well as standard, showed anti-psoriatic activity, where the PASI was found to be significantly (*p* < 0.05) declined by 45.2%, 56.5%, and 56.5%, respectively, when compared to the IMQU group. Notably, the NC of *D. tortuosa and D. triradiata* were nonsignificant from the standard group; meanwhile, all treated groups are still significant from the normal control group (Fig. 4).

#### *D. tortuosa* and *D. triradiata* NC suppressed systemic adverse effects in IMQU-induced psoriatic lesions in mice

**Fig. 5:**
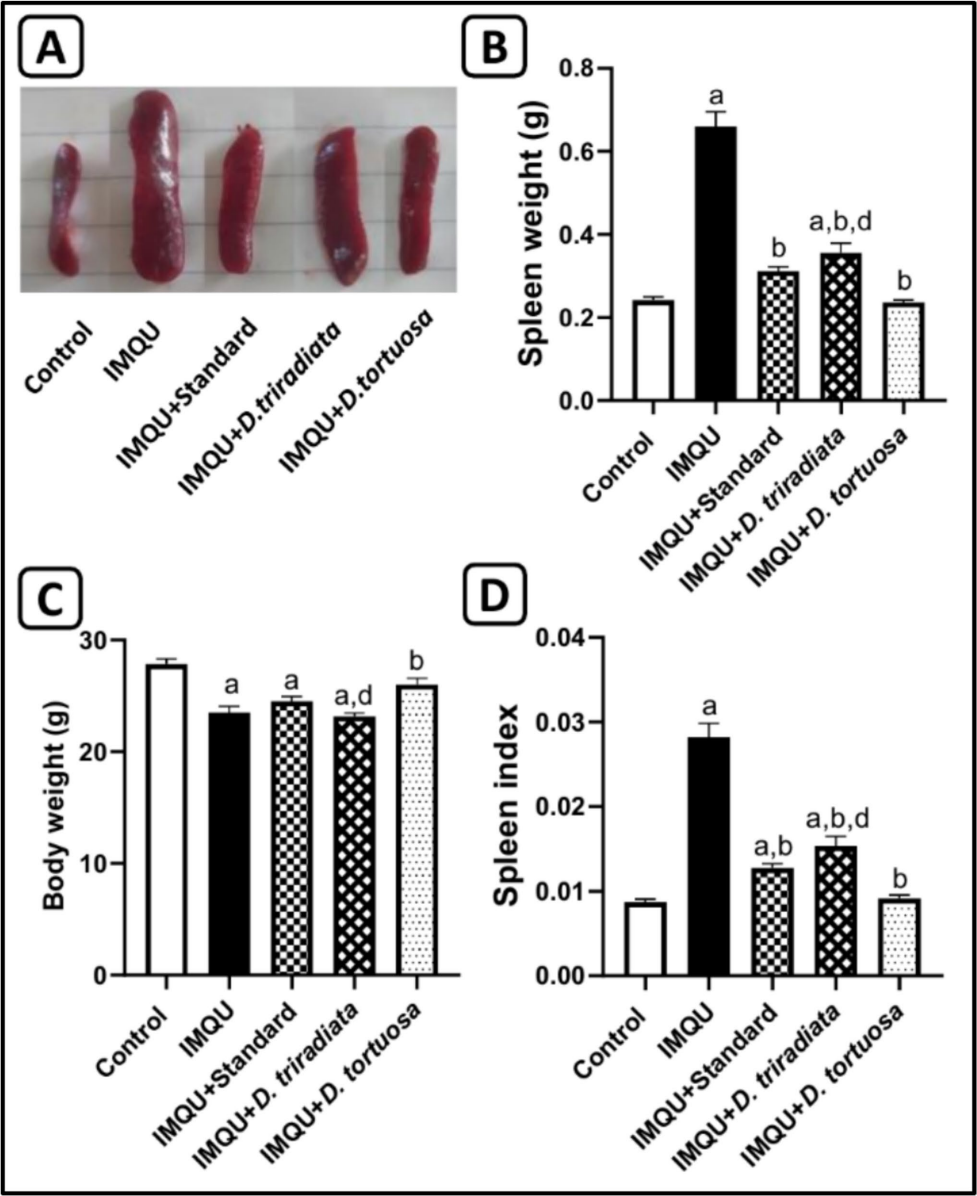
***D. tortuosa* and *D. triradiata* NC ameliorated systemic side effects in IMQU-induced psoriasis in mice indicated by A) The macroscopic image of the spleen, B) Spleen weight, C) Body weight, and D) Spleen index.** Data expressed as M ± SE, (n = 6). The test for significance was performed using ANOVA test followed by Tukey’s post-hoc test (*p* < 0.05). The significance was expressed using the following symbols: a: significant from control, b: significant from IMQU, c: significant from standard, and d: significant from *D. tortuosa*

Spleen weight and spleen index, along with body weight, were estimated to evaluate the systemic adverse effects of IMQU. Topical application of IMQU resulted in a significant (*p* < 0.05) decline in body weight by 15.6% and a significant (*p* < 0.05) 2.7-fold increase in spleen weight and 3.2-fold increase in spleen index, compared to the control group. However, the NC of *D. triradiate, D. tortuosa,* as well as the standard, led to a significant change in these parameters, with a significant (*p* < 0.05) increase in body weight by 0.99-fold, 1.11-fold, and 1.04-fold, respectively; and a significant (*p* < 0.05) decrease in spleen weight by 46.2%, 64.1%, and 52.8%, respectively, and the spleen index by 45.5%, 67.5%, and 54.8%, respectively, compared to the IMQU group. Remarkably, the greatest effectiveness was seen in the group treated with *D. tortuosa,* which showed a nonsignificant difference from the control group (Fig. 5).

#### *D. tortuosa* and *D. triradiata* NC suppressed inflammatory cytokine in IMQU-induced psoriatic lesions

**Fig. 6:**
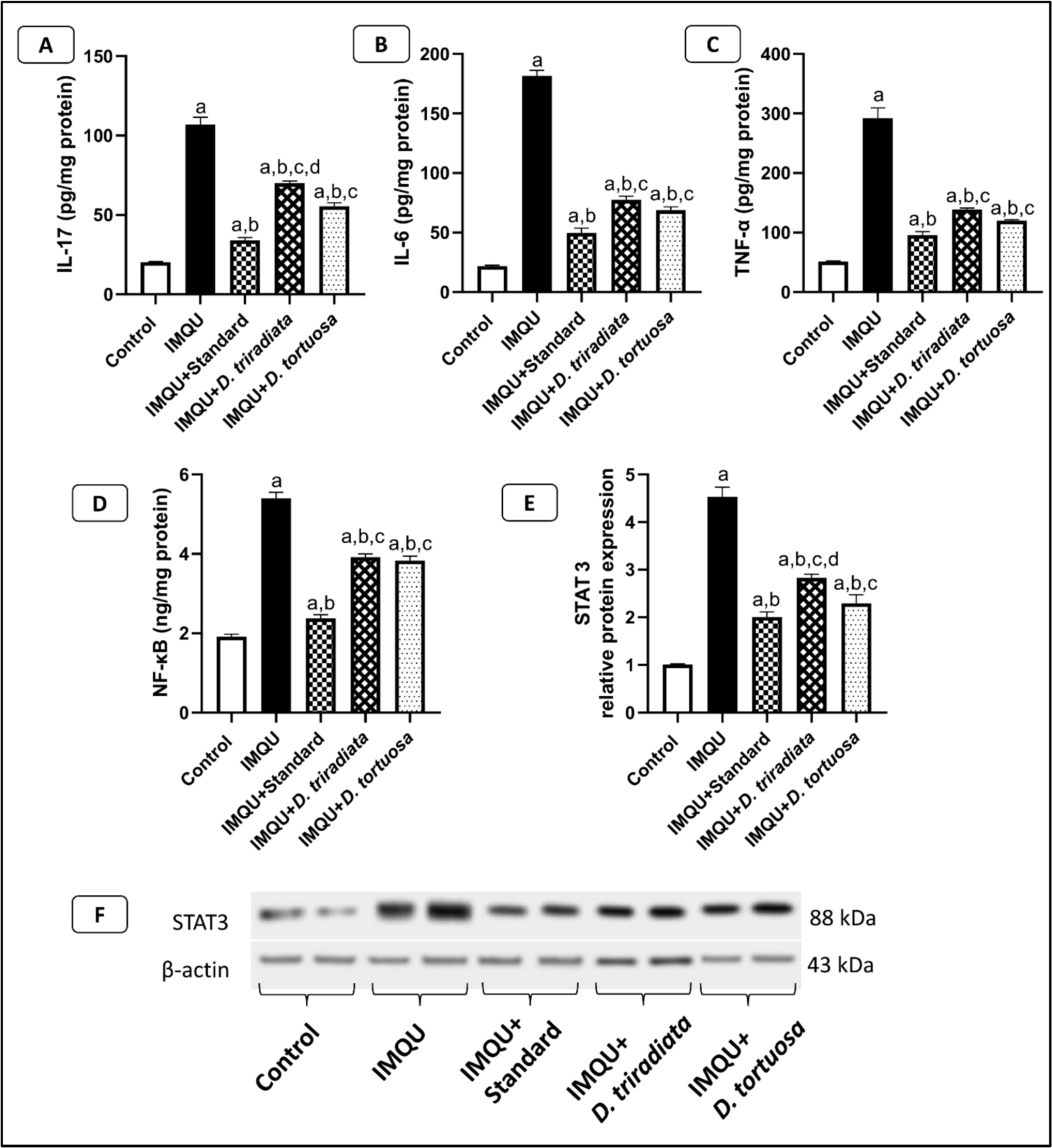
***D. tortuosa* and *D. triradiata* NC ameliorated skin A) IL-17, B) IL-6, C) TNF-α, D) NF-κB, and E) GSK-3β, in IMQU-induced psoriasis in mice.** Data expressed as M ± SE, (n = 6), The test for significance was performed using ANOVA test followed by Tukey’s post-hoc test (*p* < 0.05). The significance was expressed using the following symbols: a: significant from control, b: significant from IMQU, c: significant from standard, and d: significant from *D. tortuosa*

As shown in Fig. 6A, B, and C, the levels of inflammatory cytokines, including IL-17, IL-6, and TNF-α, were significantly (*p* < 0.05) elevated following the topical application of IMQU by 5.3-fold, 8.3-fold, and 5.7-fold, respectively, compared to the control group. Treatment with the standard and NC of *D*. *triradiate*, *D*. *tortuosa,* and the standard significantly (*p* < 0.05) reduced the level of IL-17 by 34.6%, 48.2%, and 68.1%, respectively, and the level of IL-6 by 57.3%, 62.0%, and 72.6%, respectively; and the level of TNF-α by 52.5%, 59.0%, and 67.2%, respectively, compared to the IMQU group. Notably, mice treated with *D. tortuosa* exhibited a significant (*p* < 0.05) decrease in IL-17 by 20.8% compared to those treated with *D*. *triradiate*. All cytokine levels in the standard group were significantly (*p* < 0.05) lower than those in the *D*. *triradiata* and* D*. *tortuosa* groups. All treatments are still significantly (*p* < 0.05) different from the normal control group.

#### *D. tortuosa* and *D. triradiata* NC declined NF-κB in IMQU-induced psoriatic lesions

The IMQU group exhibited a significant (*p* < 0.05) 2.8-fold increase in NF-κB levels compared to the normal control group. Meanwhile, topical application of *D*. *tortuosa*, *D*. *triradiata* NC, and the standard treatment significantly lowered NF-κB levels by 27.5%, 29.1%, and 56.1%, respectively, compared to the IMQU group. The level of NF-κB in the standard group was significantly lower than *D*. *tortuosa*, *D*. *triradiata* NC All treatments are still significantly different from the normal control group (Fig. 6D).

#### *D. tortuosa* and *D. triradiata* NC declined STAT3 in IMQU-induced psoriatic lesions

Western blotting assay was carried out to quantify STAT3 protein expression in the skin tissues. The level of STAT3 was significantly increased in the IMQU-treated group by 4.5-fold, compared to the control group. Conversely, topical application of *D*. *triradiata* and *D*. *tortuosa* NC, along with the standard cream, significantly reduced the protein expression of STAT3 by 37.5%, 49.4%, and 55.7%, respectively, compared to the IMQU-treated group.

Notably, mice treated with *D. tortuosa* exhibited a significant (*p* < 0.05) decrease in STAT3 protein expression by 18.9% compared to those treated with *D*. *triradiate*; meanwhile, both treatments are significant (*p* < 0.05) from the standard group. All treatments are still significantly (*p* < 0.05) different from the normal control group (Fig. 6E, F).

#### *D. tortuosa* and *D. triradiata* NC suppressed the expression of Ki67 in IMQU-induced psoriatic lesions

**Fig. 7:**
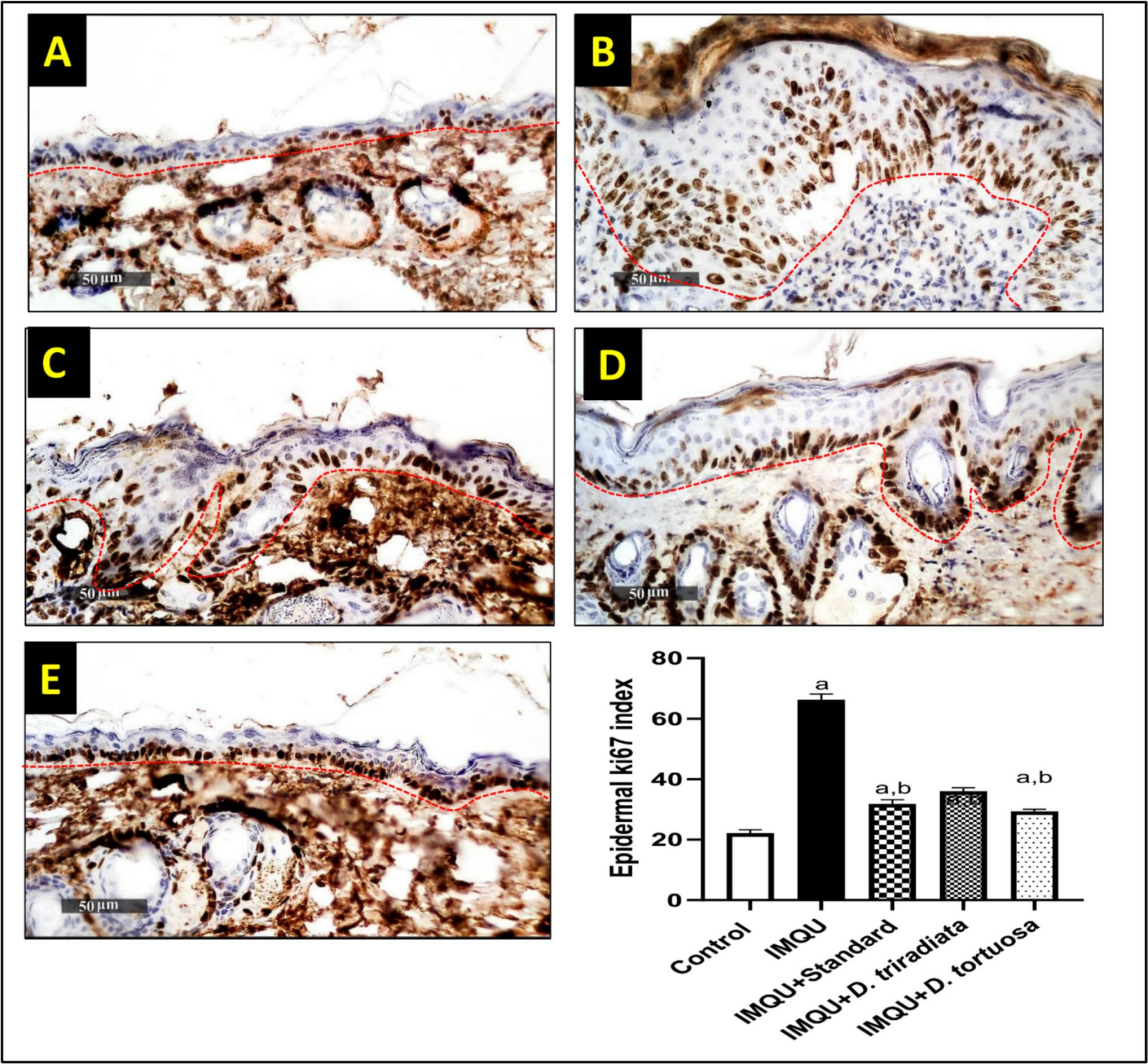
***D. tortuosa* and *D. triradiata* NC decreased the immunohistochemical expression level of epidermal Ki67 in IMQU-induced psoriasis in mice, indicated by the imaging of A) Normal control, B) IMQU, C) IMQU + Standard, D) IMQU + *D. triradiata* and E) IMQ + *D. tortuosa*. The graph represents the quantitative measurement of the epidermal Ki67 index.** Data expressed as M ± SD, (n = 6). The test for significance was performed using ANOVA test followed by Tukey’s post-hoc test (*p* < 0.05). The significance was expressed using the following symbols: a: significant from control, b: significant from IMQU, c: significant from standard, and d: significant from *D. tortuosa*. 400X. The dashed red line marks the epidermal basement membrane and subepidermal zone

Immunohistochemical expression of the mean epidermal Ki67 index demonstrated a fourfold increase in IMQU model samples compared to the normal control group. However, a marked decrease in the mean Ki-67 expression up to 52% in standard-treated samples compared with IMQU samples. *D. triradiata* and *D. tortuosa* NC demonstrated a 45.7% and 55.8% decrease in the mean Ki-67 index compared with IMQU samples. All treatments are still significantly different from the normal control group (Fig. 7).

#### *D. tortuosa* and *D. triradiata* NC inhibited the histological deterioration in IMQU-induced psoriatic lesions

**Fig. 8:**
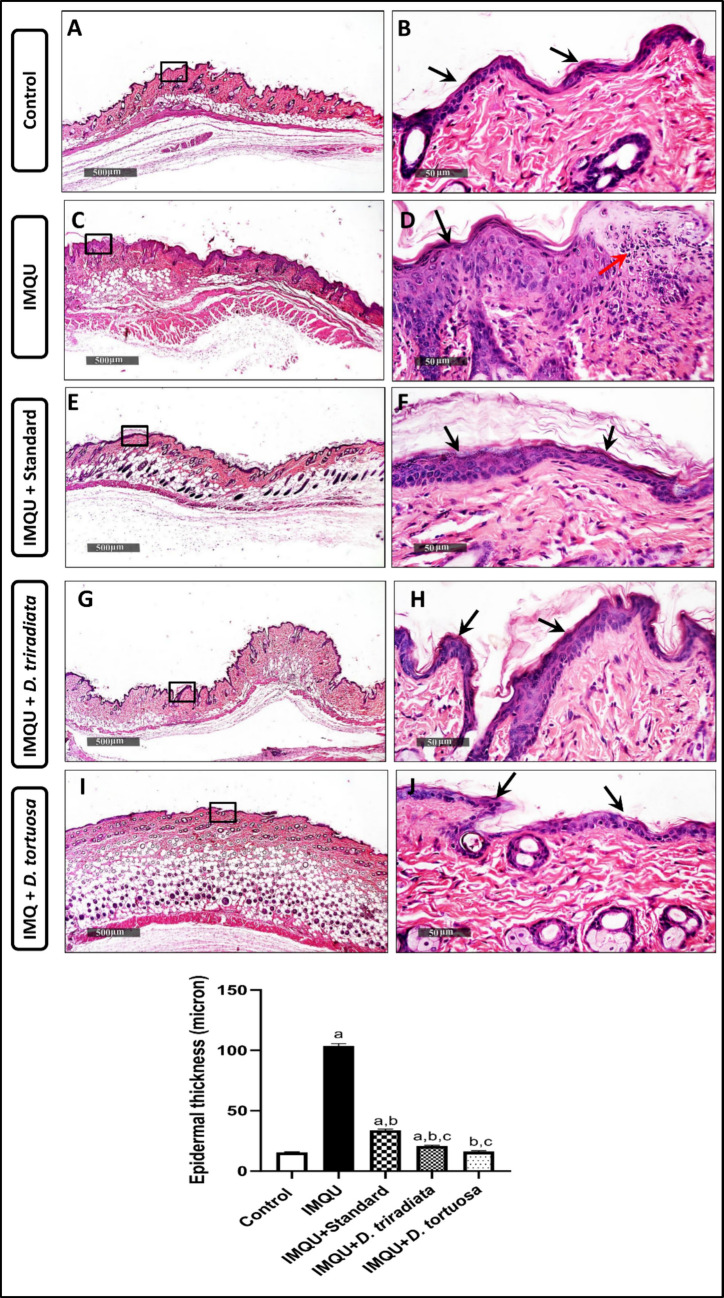
***D. tortuosa and D. triradiata NC* inhibited the histological deterioration in IMQU-induced psoriatic lesions in mice, and the mean morphometric analysis of epidermal layer thickness. The imaging represent: Control group (A&B), IMQU group (C&D), Standard group (E&F), *D. triradiata* group (G&H), and *D. tortuosa*** **(I&J) **Epidermis (black arrow), microabscess (red arrow) H&E stain, 40X & 400X. **The graph represents the quantitative measurement of the epidermal Thickness.** Data expressed as M ± SD, (n = 6). The test for significance was performed using ANOVA test followed by Tukey’s post-hoc test (*p* < 0.05). The significance was expressed using the following symbols: a: significant from control, b: significant from IMQU, and c: significant from standard

Normal, organized histological features of the skin layers in control samples showed a mean epidermal thickness of up to 9.6 µm. Additionally, an apparently intact dermal layer (Fig. 8A, B) with normally organized collagen fibers was observed. However, a 6.7-fold increase in epidermal thickness was observed in the IMQU-treated group compared to the controls, accompanied by multiple microabscesses and moderate inflammatory cell infiltrates (Fig. 8C, D). A significant decrease in epidermal thickness, up to 67.4%, was recorded in the standard-treated samples compared to the IMQU group, with minimal signs of inflammation (Fig. 8E, F). Furthermore, higher protective efficacy was observed in *D*. *tortuosa* and *D*. *triradiata* groups, which showed lower epidermal thickening—up to 84.4% and 79.9% decreases, respectively, compared to the IMQU group (Fig. 8G-J). All treatments are still significantly different from the normal control group.

## Discussion

Psoriasis is a chronic autoimmune skin condition characterized by inflammation, excessive cytokine production, and a compromised skin barrier, leading to symptoms such as redness, scaling, and thickening (Chen et al. 2022). Current treatments, including topical therapies, systemic medications, and phototherapy, have resulted in clinical improvements. However, these options are mainly reserved for severe cases due to their toxicity, and the long-term use of synthetic or biological agents can cause side effects and incur high costs. Topical treatments are typically preferred because of their lower cost, improved patient compliance, and targeted delivery to affected skin areas (Pukale et al. 2021; Sala et al. 2016). Consequently, researchers are exploring topical applications of medicinal plants to reduce the side effects and limitations of conventional or biological therapies (Ben-Arye et al. 2003). Polyphenols exert multiple mechanisms in the treatment of psoriasis, serving as antioxidants and anti-inflammatory agents (Di Salvo et al. 2023). However, their use is often hindered by sensitivity to environmental conditions and low bioavailability (Shah and Williams 2014). Recently, NEs have been proposed to address these challenges and enhance the pharmacological effects of polyphenols (Nichols and Katiyar 2010). Several reports indicate that these NEs can serve as highly effective transdermal vehicles (Rai et al. 2018) and demonstrate significant anti-psoriatic activity (Salim et al. 2016) when used to deliver drugs for the treatment of psoriasis. These are homogeneous and transparent solutions composed of oil, surfactant, cosurfactant, and water in specific ratios, typically used as carriers for insoluble, hydrophobic, or hydrolysable compounds. Thus, we developed NE and incorporated it into a cream formula to give NC. The prepared NC can enhance the solubility of DAEE and provide a more efficient method for improving its biodistribution at inflamed skin sites in psoriasis patients. Therefore, NC for the *D*. *tortuosa* and *D*. *triradiata* extracts was used to enhance their anti-psoriatic effects. Previous reports have demonstrated that the aqueous ethanol extract of *D. tortuosa's* aerial parts exhibits antioxidant and antimicrobial properties (Ahmed et al. 2021). Additionally, the antioxidant activity of *D*. *triradiata* shoot extract has been evaluated (Elmosallamy et al. 2021). The phenolic compounds in *D*. *tortuosa* and *D*. *triradiata* are responsible for their biological effects.

This study evaluated the skin-barrier-protective and anti-inflammatory effects of NCs of DAEE extracted from the aerial parts of *D*. *tortuosa* and *D*. *triradiata,* using biochemical and histopathological parameters, to comprehensively assess their anti-psoriatic efficacy. The treatment was compared to tacrolimus, a standard drug. Tacrolimus is a calcineurin inhibitor used occasionally to treat various immune-mediated inflammatory skin conditions, such as atopic dermatitis and psoriasis. It works by selectively blocking T cell activation, leading to anti-inflammatory and immunosuppressive effects (Fereig et al. 2021; Park et al. 2020). These effects support our findings, as the group treated with tacrolimus showed significant improvements in the measured parameters compared to the IMQU group. A marked reduction in PASI scores was observed in the treated groups compared to the psoriasis control, indicating a visible improvement in erythema, scaling, and skin thickening. These clinical improvements align with previous findings (Tawfik et al. 2024; Xu et al. 2022) that associate PASI reduction with effective anti-inflammatory therapy in psoriasis models. Psoriasis is often accompanied by systemic immune activation, indicated by spleen hypertrophy. Psoriatic inflammation is known to trigger splenomegaly due to heightened immune cell proliferation and cytokine signaling (Brunner et al. 2023; Hjuler et al. 2018). Treatment with the NC of DAEE notably reduced both spleen weight and spleen index, suggesting a reduction in systemic inflammation. These findings support the idea that localized skin treatment can have indirect immunomodulatory activity, possibly due to reduced antigenic stimulation and cytokine release. Pro-inflammatory cytokines such as TNF-α, IL-6, and IL-17 are central to the pathogenesis of psoriasis, mediating keratinocyte proliferation and immune cell recruitment (Kjær et al. 2015)**.** Both formulations significantly reduced levels of these cytokines, highlighting their anti-inflammatory potential. IL-17, in particular, plays a critical role in psoriatic plaque formation, and its downregulation is indicative of a targeted therapeutic effect as documented in our results. The NF-κB and STAT3 pathways regulate the expression of pro-inflammatory genes and are hyperactivated in psoriatic lesions. NF-κB plays a crucial role in initiating and sustaining inflammation by promoting the transcription of pro-inflammatory genes (Goldminz et al. 2013)**.** This finding is supported by elevated levels of inflammatory cytokines, including TNF-α, IL-6, and IL-17. Signal transducer and activator of transcription 3 (STAT3) plays a crucial role in the pathogenesis of psoriasis. STAT3 is critically involved in keratinocyte hyperproliferation and Th17 cell differentiation (Calautti et al. 2018; Sano et al. 2005)**.** Miyoshi et al. demonstrated that STAT3 inhibition effectively suppresses the manifestation of psoriatic phenotypes in both transgenic mice and in individuals diagnosed with psoriasis (Miyoshi et al. 2011). The significant suppression of both transcription factors suggests that the NCs of DAEE exert their effects by inhibiting key intracellular signaling pathways, thereby attenuating both the inflammatory and proliferative components of the disease. This suppression may underlie the observed reduction in cytokines and clinical improvement. Additionally, immunohistochemical analysis of Ki67, a marker of cellular proliferation, showed significantly decreased expression in the epidermis of treated groups. In psoriasis, Ki67 overexpression reflects uncontrolled keratinocyte proliferation (Pukale et al. 2021). Its suppression following treatment indicates a return toward normal epidermal cell turnover, further validating the antiproliferative potential of the NCs of DAEE, and this complements the reduced PASI scores of the treated groups.

Histopathological analysis corroborated the biochemical and clinical findings. Psoriatic skin that was not treated displayed classic characteristics, including epidermal hyperplasia and a dense inflammatory infiltrate in the dermis. In contrast, these characteristics were significantly diminished in the treatment groups, with skin sections nearing normal histological structure. The decrease in epidermal layer thickness and inflammatory cell infiltration validates the therapeutic effectiveness of the formulations at the tissue level. The combined clinical and histological evidence strongly supports the anti-psoriatic efficacy of the DAEE NCs. Their ability to modulate essential inflammatory and proliferative pathways, along with systemic immune markers, indicates their potential as effective topical treatments. Further research, including human trials and mechanistic analyses, is necessary to verify their translational viability. Psoriasis is characterized by an overactive immune response, leading to rapid skin cell proliferation and elevated inflammatory cytokine levels. Polyphenols are being investigated for their potential to reduce psoriasis symptoms through their anti-inflammatory and immune-modulatory properties, which help modulate the immune response. Plant-based polyphenols may affect psoriasis by reducing the production of pro-inflammatory cytokines such as TNF-α, IL-6, and IL-17. and blocking NF-κB. Additionally, the immunomodulatory effects of polyphenols enhance the immune response by regulating miRNAs in keratinocytes, which are associated with various outcomes, including altered STAT3 levels (Kocic et al. 2019). Vanillic acid, the major phenolic acid in *D. tortuosa* and *D. triradiata*, is noted for its anti-inflammatory properties through the inhibition of oxidative stress, pro-inflammatory cytokine production, and NF-κB activation (Calixto-Campos et al. 2015). Furthermore, rutin, a significant flavonol glycoside, is well documented to be effective in treating various inflammatory diseases, including psoriasis (Lang and Han 2022; Shandil et al. 2020). It achieves its effects by inhibiting IL-6 and TNF-α and by blocking the JAK2/STAT3 signaling pathway, thereby diminishing psoriasis-related inflammation and keratinocyte dysdifferentiation. Additionally, the anti-psoriatic activity of both extracts may stem from the synergistic effects of the phenolic compounds present in DAEE and other constituents of the extract, which may aid in the treatment of psoriasis, rather than from a single major component.

## Conclusion

In conclusion, the present study highlights the promising anti-psoriatic potential of NC loaded with DAEE of *D. tortuosa* and *D. triradiata*. These formulations demonstrated significant clinical, biochemical, and histopathological improvements in an IMUQ-induced psoriasis model, comparable to the standard drug tacrolimus. The observed therapeutic effects are attributed to the polyphenolic content, particularly vanillic acid and rutin, which exert anti-inflammatory and immunomodulatory actions by suppressing key cytokines, inhibiting the NF-κB and STAT3 pathways, and reducing keratinocyte hyperproliferation, as indicated by reduced Ki-67 expression. Additionally, the NCs effectively improved skin barrier integrity and mitigated systemic inflammation, as evidenced by reduced spleen hypertrophy. These findings reinforced the use of DAEE-loaded NCs as a potential topical therapeutic strategy for psoriasis. However, further investigations, including pharmacokinetic profiling, mechanistic studies, and clinical trials, are warranted to validate their efficacy and safety in human applications.

## Supplementary Information

Below is the link to the electronic supplementary material.

Supplementary file1 (DOCX 29 KB)

## Data Availability

The data supporting the findings of this study are included within the article and its supplementary materials.

## Abbreviations

*D. tortuosa*: *Deverra tortuosa*
*D. triradiata*: *Deverra triradiata*
DAEE: Defatted aqueous ethanol extract
2D-PCE: two-dimensional paper chromatography
IL: Interleukins
IMQ: Imiquimod
NC: Nanocream
NE: Nanoemulsion
NE: Nanoemulsions
NF-κB: Nuclear factor kappa B
PASI: Psoriasis area and severity index
STAT-3: Signal transducer and activator of transcription 3
TNF-α: Tumor necrosis factor-alpha
