# Supplementary material for: Anti-psoriatic potential of nanocarrier formulated with Deverra tortuosa DC. and Deverra triradiata hochst aerial extracts: in vivo evaluation in mice
Source: Saudi Pharm J. 2026 May 26;34(3):31. doi: 10.1007/s44446-026-00086-y (PMC13212808; doi:10.1007/s44446-026-00086-y)
Supplement: Supplementary file 1 — Supplementary file1 (DOCX 29 KB) [file 44446_2026_86_MOESM1_ESM.docx]

**HPLC-MS/MS condition**

Quantification of the phenolic content was done by a modified version previously mentioned,^24^ . Agilent 1290 Infinity series with triple quadrupole 6420 HPLC-MS/MS (Agilent Technology, Santa Clara, California, United States), coupled with an ESI source, worked in -ve and +ve ionization approaches, was used. Flow injection analysis (FIA) was used for the optimization of the MS/MS parameters of standards. Phenolic components were separated by injecting the diluted samples (1:5) into the Phenomenex Synergi Polar-RP C18 column (250 x 4.6 mm, 4 µm). The mobile system is water and MeOH for solvent A and B respectively, each one containing formic acid (0.1 %) as well as, its preparation being isocratic mode, 20 % B (0 -0.1 min); gradient mode, 20 - 85 % B (1-25 min); isocratic mode, 85% B (25-26 min); gradient mode, 85-20% B (26–32 min), The column and the drying gas temperature in the ionization source were adjusted at 30 and 350 °C respectively. The flow rate of the gas and injected volume (2 μL) is 12 L and 0.8 mL min^-1,^ respectively, as well as the voltage of the capillary and nebulizer pressure were 4000 V and 55 psi, respectively. For quantitation, the peak area was integrated after recognition in the dynamic-multiple reaction monitoring (dynamic-MRM) mode then for quantitation, then the maximum production was used, while for qualitative analysis, the remaining ions were used. The retention time of each compound was established at 2 min

**Development of pseudo-ternary phase diagram for both extracts:**

It was established based on our previously published work [49]. In brief, oleic acid and jojoba oil (1:1 w/w) served as the oily phase, and polysorbate 80 with propanol alcohol (surfactant/cosurfactant) were mixed in various ratios (0.5:3.5, 1:3, 1.5:2.5, 2:2, 2.5:1.5, 3:1, 3.5:0.5, 0.5:4.5, 1:4, 1.5:3.5, 2:3, 2.5:2.5, 3:2, 3.5:1.5, 4:1, and 4.5:0.5 w/w). The oily phase and surfactant/cosurfactant at ratios of 1:4, 1.5:2.5, 2:3, and 2.5:2.5 were mixed using a hot plate stirrer, then titrated with deionized water until stable turbidity was achieved. All weights were recorded and calculated as w/w %, and the pseudo-ternary phase diagram was created using Chemix school ternary diagram software.

**Table (S1): Composition of oils phase, surfactant/co-surfactant, aqueous phase and *Deverra* extracts**

| Formulae  /components | Oil phase w/w%  (Oleic/Jojoba) | SAA/Co-SAA w/w% | Water w/w% | *D*. *tortuosa extract*  w/w% | *D. triradiata extract*  w/w% |
| --- | --- | --- | --- | --- | --- |
| F1 | 6.3 | 31.3 | 62.4 | - | - |
| F2 | 6.3 | 31.3 | 54.4 | 8 | - |
| F3 | 6.3 | 31.3 | 54.4 | - | 8 |

**Table (S2) Composition of the prepared o/w vanishing cream containing nanoemlusion**

| Contents (w/w%) | F1 | F2 | F3 |
| --- | --- | --- | --- |
| Nanoemulsion with the extracts | 25  Plain nanoemlusion | *25*  Nanoemulsion with *D. tortuosa* | *25*  Nanoemulsion with  *D. triradiata* |
| Stearic acid | 10 | 10 | 10 |
| White soft paraffin | 5 | 5 | 5 |
| Liquid paraffin | 5 | 5 | 5 |
| Polysorbate 80 | 1 | 1 | 1 |
| Propylene glycol | 8 | 8 | 8 |
| Water | 46 | 46 | 46 |

**Table S3: HPLC–MS/MS** **acquisition parameters (dynamic-MRM mode) used for analysis of 38 marker compounds.**

| Compounds | Precursor  ion, *m/z* | Product  ion, *m/z* | Fragmentor, V | Collision energy, V | Polarity | R_t_ |
| --- | --- | --- | --- | --- | --- | --- |
| Gallic acid | 169 | 125.2^*^ | 97 | 12 | Negative | 6.96 |
| Neochlorogenic acid | 353 | 191.2^*^, 179 | 82 | 12, 12 | Negative | 9.52 |
| Delphinidin-3-galactoside | 465.01 | 303^*^ | 121 | 20 | Positive | 11.36 |
| (+)-Catechin | 289 | 245.2^*^,109.2 | 131 | 8, 20 | Negative | 11.44 |
| Procyanidin B2 | 576.99 | 576.99^*^, 321.2 | 160 | 0, 32 | Negative | 12.41 |
| Chlorogenic acid | 353 | 191.2^*^, 127.5 | 82 | 12, 20 | Negative | 12.42 |
| *p*-Hydroxybenzoic acid | 137 | 93.2^*^ | 92 | 16 | Negative | 12.86 |
| (-)-Epicatechin | 289 | 245.1^*^, 109.1 | 126 | 8, 20 | Negative | 13.03 |
| Cyanidin-3-glucoside | 449 | 287.3^*^, 255.6 | 121 | 20, 20 | Positive | 13.14 |
| Petunidin-3-glucoside | 479.01 | 317^*^, 302 | 121 | 20, 44 | Positive | 13.26 |
| 3-Hydroxybenzoic acid | 137 | 93.2^*^ | 88 | 8 | Negative | 13.59 |
| Caffeic acid | 179 | 135.2^*^, 134.1 | 92 | 12, 24 | Negative | 13.65 |
| Vanillic acid | 167 | 152.4^*^, 108.1 | 88 | 12, 20 | Negative | 14.32 |
| Resveratrol | 227 | 185^*^, 143.2 | 136 | 12, 20 | Negative | 14.40 |
| Pelargonidin-3-glucoside | 433.01 | 271^*^, 121 | 116 | 24, 50 | Positive | 14.52 |
| Pelagonidin-3-rutinoside | 579.01 | 271^*^ | 145 | 32 | Positive | 14.56 |
| Malvidin-3-galactoside | 493.01 | 331^*^, 315.1 | 121 | 20, 50 | Positive | 14.64 |
| Syringic acid | 196.9 | 182.2^*^, 121.2 | 93 | 8, 12 | Negative | 15.28 |
| Procyanidin A2 | 575 | 575^*^, 285 | 170 | 0, 20 | Negative | 16.18 |
| *p*-Coumaric acid | 163 | 119.2^*^, 93.2 | 83 | 12, 36 | Negative | 16.70 |
| Ferulic acid | 193 | 134.2^*^, 131.6 | 83 | 12, 8 | Negative | 17.10 |
| 3,5-Dicaffeoylquinic acid | 514.9 | 353.1^*^, 191 | 117 | 8, 28 | Negative | 17.61 |
| Rutin | 609 | 300.2^*^, 271.2 | 170 | 32, 50 | Negative | 17.73 |
| Hyperoside | 465.01 | 303^*^, 61.1 | 97 | 8, 50 | Positive | 18.33 |
| Isoquercitrin | 463 | 271.2^*^, 300.2 | 155 | 44, 24 | Negative | 18.36 |
| Delphinidin-3,5-diglucoside | 462.9 | 300.1^*^ | 165 | 24 | Negative | 18.38 |
| Phloridzin | 435.39 | 273^*^, 167 | 155 | 8, 28 | Negative | 18.83 |
| Quercitrin | 446.99 | 300.2^*^, 301.2 | 160 | 24, 16 | Negative | 19.61 |
| Myricetin | 316.99 | 179.1^*^, 182 | 150 | 16, 24 | Negative | 19.61 |
| Naringin | 578.99 | 271.3^*^, 151.3 | 170 | 32, 44 | Negative | 19.62 |
| Kaempferol-3-glucoside | 447 | 284.2^*^, 255.2 | 170 | 24, 40 | Negative | 19.77 |
| Hesperidin | 611.01 | 303^*^, 334.8 | 112 | 20, 12 | Positive | 20.19 |
| Ellagic acid | 301 | 301^*^, 229 | 170 | 0, 24 | Negative | 21.41 |
| *Trans*-Cinnamic acid | 149 | 131.2^*^, 77.2 | 74 | 4, 36 | Positive | 21.44 |
| Quercetin | 300.99 | 151.2^*^, 179.2 | 145 | 16, 12 | Negative | 21.87 |
| Phloretin | 272.99 | 167^*^, 123 | 116 | 8, 20 | Negative | 22.30 |
| Kaempferol | 287.01 | 153^*^, 69.1 | 60 | 36, 50 | Positive | 23.84 |
| Isorhamnetin | 314.99 | 300.2^*^, 196.1 | 145 | 16, 4 | Negative | 24.57 |

R_t_ Retention time in min; ^*^These product ions were used for quantification.

Separation condition: The mobile system, water (solvent A) and methanol (solvent B), both with 0.1% formic acid, was prepared and used as follows: 0–1 min in isocratic mode, 20% B; 1–25 min, gradient mode, 20–85% B; 25–26 min, isocratic mode, 85% B; 26–32 min, gradient mode, 85–20% B. The injection volume was 2 μl, and the flow rate was kept at 0.8 mL/min. The temperature of the column was set to 30 °C, and the drying gas temperature in the ionization source was set to 350 °C. The flow rate of the gas was adjusted to 12 L/min, the capillary voltage was 4000 V and the nebulizer pressure was 55 psi. The peak areas were integrated for quantitation after detection in the dynamic-multiple reaction monitoring (dynamic-MRM) mode. Each analyte's most abundant product ion was employed for quantification, while the other ions were used for qualitative analysis. Each compound's unique time window (Δ retention time) was set at 2 min.

**Figure S1: Calibration curve for estimation of the total phenolic content.**
